# Editorial: Composites and surface and interface engineering (CSIE): preparation and modification of biomaterials and their anti-biofouling ability and surface wettability

**DOI:** 10.3389/fbioe.2023.1230571

**Published:** 2023-06-12

**Authors:** Zhoukun He, Navid Rabiee, Qiang Wei, Yong Hou, Bin Yan, Jing Xie

**Affiliations:** ^1^ Institute for Advanced Study, Research Center of Composites and Surface and Interface Engineering, Chengdu University, Chengdu, China; ^2^ Centre for Molecular Medicine and Innovative Therapeutics, Murdoch University, Perth, WA, Australia; ^3^ School of Engineering, Macquarie University, Sydney, NSW, Australia; ^4^ College of Polymer Science and Engineering, State Key Laboratory of Polymer Materials and Engineering, Sichuan University, Chengdu, China; ^5^ Department of Electrical and Electronic Engineering, The University of Hong Kong, Hong Kong, Hong Kong SAR, China; ^6^ College of Biomass Science and Engineering, Sichuan University, Chengdu, China; ^7^ Institute of Biomedical Engineering, West China School of Basic Medical Sciences and Forensic Medicine, Sichuan University, Chengdu, China

**Keywords:** composites, biomaterials, antifouling, surface and interface, surface wettability

In recent years, polymer and metal composites have been extensively explored as implanted or interventional biomaterials and medical devices. When the surface of a biomaterial comes into contact with cells, proteins, bacteria, etc., their interaction can have a serious impact on the performance of the biomaterial itself. For example, when biomaterials are used as scaffolds for tissue engineering, their good biocompatibility will enhance the proliferation of desired cells and accelerate tissue repair. In this situation, good cell adhesion is required. However, when biomaterials are used as inert materials, the adhesion of cells, proteins, or bacteria on their surface will form dense collagenous capsules around the implanted or interventional biomaterials and medical devices, which would induce inflammatory responses and may lead to infection and/or implant rejection. In this situation, anti-fouling ability is essential. Therefore, the interaction between the biomaterial and cells, proteins, bacteria, etc., which is affected by the surface wettability of the biomaterial, should be well controlled.

Polycaprolactone (PCL), as a common synthetic biodegradable polymer, which takes a long time to break down in the human body, can be used as an implanted scaffold for bone formation. Al-Bishari et al. reported a multifunctional PCL nanofiber membrane loaded with vitamin D (Vit D) and curcumin (Cur) to accelerate osteogenesis by electrospinning technology. Because of the hydrophobicity of PCL and Vit D, the authors modified Vit D with hydrophilic polyethylene glycol, and finally a hydrophilic PCL/Vit D-Cur membrane with a water contact angle (WCA) of 12.2° was achieved. Therefore, the biocompatibility of the PCL scaffolds and the release of Cur were improved. Meanwhile, the cell proliferation/differentiation, the vascular endothelial growth factor expression, and the bone morphogenetic protein-2 expression were also improved, indicating that this study may offer an efficient strategy for developing PCL scaffolds for bone regeneration.

Similarly, Li et al. reported a poly (L-lactide-co-ε-caprolactone) (PLCL)-based nanofiber membrane loaded with sodium tanshinone IIA sulfonate (STS) for potential vascular graft application through a coaxial electrospinning technology. PLCL was used as the shell layers, and hydrophilic polyethylene oxide (PEO) loaded with STS was used as the core layers to fabricate nanofiber membranes with oriented-fiber or random-fiber morphology. The hydrophilicity of the oriented-fiber membranes with a WCA of approximately 40° was greatly improved compared with that of the random-fiber membranes with a WCA of approximately 110°. The improved surface hydrophilicity, due to the introduction of PEO and the oriented-fiber morphology was beneficial in reducing the inflammatory response and the gradual rupture of the fibers. Meanwhile, the surface hydrophilicity related to the adhesion and cohesion of platelets could affect the migration and proliferation of cells, which demonstrated that the oriented-fiber membranes were more conducive to the formation of new tissue.

Liao et al. reported a metallic composite based on stainless steel (SS), which was modified through a piranha solution treatment with enhanced hydrophilicity, to improve the anti-biofouling (antithrombotic) ability for potential blood contact materials. After hydrophilic modification, the treated SS with a WCA of about 10° significantly reduced the adhesion and proliferation of smooth muscle cells. Therefore, the treatment of SS with piranha solution was a straightforward, simple, and efficient means to endow the surface with the anti-biofouling ability and showed potential applications in implantable medical devices.

To provide the polymer composite with excellent anti-fouling ability, He et al. conducted a systematic review of the anti-fouling applications of polydimethylsiloxane (PDMS)-based superhydrophobic materials with a WCA greater than 150° through a versatile “3M” methodology (materials, methods, and morphologies). The excellent anti-fouling ability of the superhydrophobic surface should be attributed to its fouling resistance and/or fouling release effects. To reveal the excellent anti-fouling behavior of PDMS-based superhydrophobic materials, three types of PDMS-based surfaces containing pure PDMS, PDMS with nanoparticles, and PDMS with other materials were introduced. Taking the type of PDMS with nanoparticles as a typical example, nanoparticles with various morphologies (zero-, one-, two-, and three-dimensional nanoparticles) were systematically discussed. Based on this “3M” methodology, numerous novel superhydrophobic materials based on PDMS or other polymers will be explored for anti-fouling applications in future research.

The goal of this Research Topic is to focus on Composites and Surface and Interface Engineering (CSIE), especially for biomaterials with controllable surface wettability. The results of this Research Topic can be applied to accelerate new tissue formation such as bone healing and vascular grafting based on drug-loaded polymeric nanofiber membranes with enhanced surface hydrophilicity, to improve the anti-biofouling ability based on metallic composites such as stainless steel with enhanced surface hydrophilicity, and to improve anti-fouling ability based on polymer composites with surface superhydrophobicity. This Research Topic aims to highlight the advanced achievements in the design, preparation, modification, and characterization of various biomaterials based on surface and interface engineering strategies, which will provide a guide for future research directions in these fields and become a reference relevant to the biomaterials with controllable surface wettability for their future application in implanted or interventional biomaterials and medical devices.

